# Supplementary material for: Monitoring of Paenibacillus larvae in Lower Austria through DNA-Based Detection without De-Sporulation: 2018 to 2022
Source: Vet Sci. 2023 Mar 10;10(3):213. doi: 10.3390/vetsci10030213 (PMC10054382; doi:10.3390/vetsci10030213)
Supplement: Supplementary file 1 [file vetsci-10-00213-s001.zip › vetsci-2239517-supplementary.pdf]

Supplementary Figure S1. A typical RT-PCR plot from the commercial RT-PCR kit for *Paenibacillus larvae*. Yellow curve: positive control; yellow line: negative control; green curves are different samples and the blue line is the calculated cut-off value defined by the cycler-software.

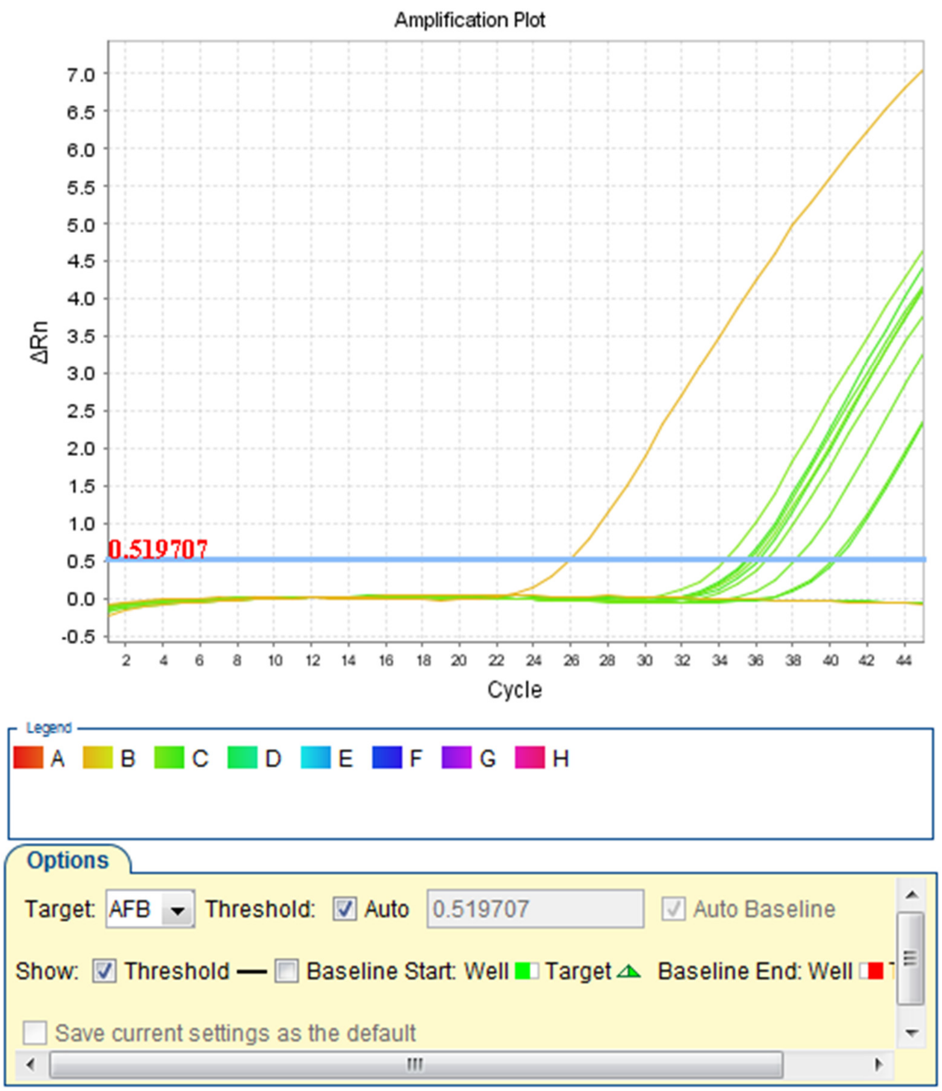

Supplementary Figure S2. Average temperatures per month, averaged from three weather stations located in the study region (Lunz am See, Oberndorf an der Melk, Wieselburg), in °C. In 2018, average temperatures in February were lower than in the following years.

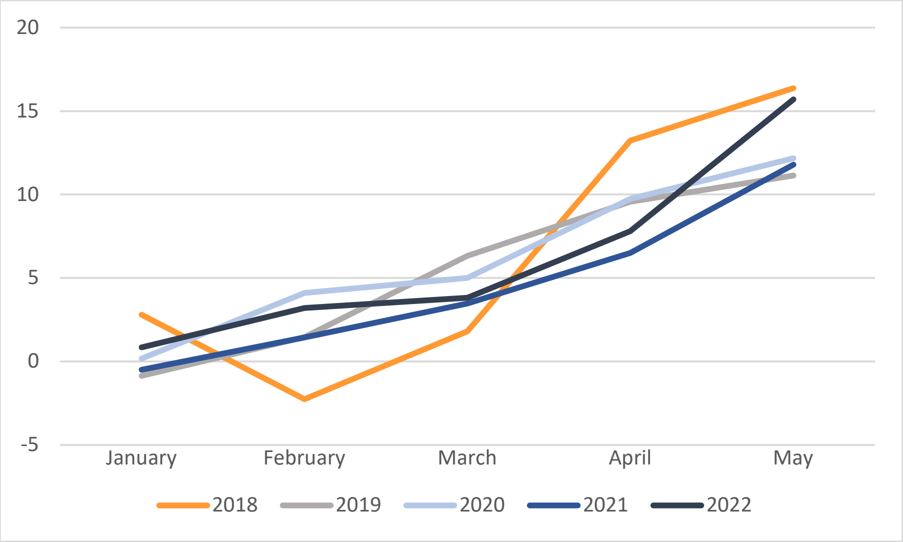

Temperature data were obtained from the Austrian weather service - Zentralanstalt für
Meteorologie und Geodynamik (https://www.zamg.ac.at/cms/en/climate/climate-overview/current_climate; last accessed 28 Feb 2023).
